# Dosimetric effects of intrafractional isocenter variation during deep inspiration breath‐hold for breast cancer patients using surface‐guided radiotherapy

**DOI:** 10.1002/acm2.12214

**Published:** 2017-11-15

**Authors:** Malin Kügele, Anneli Edvardsson, Lovisa Berg, Sara Alkner, Carina Andersson Ljus, Sofie Ceberg

**Affiliations:** ^1^ Department of Hematology, Oncology and Radiation Physics Skåne University Hospital Lund Sweden; ^2^ Medical Radiation Physics Department of clinical sciences Lund University Lund Sweden

**Keywords:** breath hold, intrafractional isocenter variation, optical surface scanning, treatment planning

## Abstract

The aim of this study was to investigate potential dose reductions to the heart, left anterior descending coronary artery (LAD), and ipsilateral lung for left‐sided breast cancer using visually guided deep inspiration breath‐hold (DIBH) with the optical surface scanning system Catalyst™, and how these potential dosimetric benefits are affected by intrafractional motion in between breath holds. For both DIBH and free breathing (FB), treatment plans were created for 20 tangential and 20 locoregional left‐sided breast cancer patients. During DIBH treatment, beam‐on was triggered by a region of interest on the xiphoid process using a 3 mm gating window. Using a novel nonrigid algorithm, the Catalyst™ system allows for simultaneous real‐time tracking of the isocenter position, which was used to calculate the intrafractional DIBH isocenter reproducibility. The 50% and 90% cumulative probabilities and maximum values of the intrafractional DIBH isocenter reproducibility were calculated and to obtain the dosimetric effect isocenter shifts corresponding to these values were performed in the treatment planning system. For both tangential and locoregional treatment, the dose to the heart, LAD and ipsilateral lung was significantly reduced for DIBH compared to FB. The intrafractional DIBH isocenter reproducibility was very good for the majority of the treatment sessions, with median values of approximately 1 mm in all three translational directions. However, for a few treatment sessions, intrafractional DIBH isocenter reproducibility of up to 5 mm was observed, which resulted in large dosimetric effects on the target volume and organs at risk. Hence, it is of importance to set tolerance levels on the intrafractional isocenter motion and not only perform DIBH based on the xiphoid process.

## 1 INTRODUCTION

Adjuvant radiotherapy for breast cancer reduces the risk of locoregional recurrence as well as breast cancer death.^1^, ^2^ However, some radiation is inevitably delivered to normal tissue, such as the heart and lungs, which has been shown to increase the risk of cardiovascular and pulmonary disease.^3^, ^4^, ^5^, ^6^, ^7^, ^8^, ^9^ Darby et al.^5^ have shown that the relative risk of ischemic heart disease increases with 7.4% per Gy increased mean heart dose, with no apparent threshold. This relationship was recently validated by van den Bogaard et al.^8^ for more modern radiotherapy techniques. Also, a higher incidence of coronary artery disease has been observed for the left anterior descending coronary artery (LAD) for left‐sided compared to right‐sided breast radiotherapy.^10^ This could possibly reduce the survival benefit of breast cancer radiotherapy.

Since a large proportion of the breast cancer patients are cured from their disease and hence become long‐term survivors, with the 5‐year survival being approximately 90%,^11^ it is important to reduce the late side‐effects as much as possible. Therefore, there has been much focus in the last years in breast cancer radiotherapy to develop treatment techniques that reduce the dose to normal tissues, such as treatment during deep inspiration, prone patient positioning, intensity modulated radiotherapy, proton radiotherapy, and partial breast radiotherapy.^12^ Treatment during deep inspiration has been shown to decrease the cardiopulmonary doses without compromising target coverage, due to increased spatial distance between the organs at risk and the target as well as decreased lung density.^13^, ^14^, ^15^, ^16^ Treatment in deep inspiration breath‐hold (DIBH) requires patient compliance and the use of visual guidance has been shown to improve intrafractional reproducibility of the inspiration level.^17^, ^18^ Several techniques for tracking the breathing motion have been introduced in radiotherapy, such as measuring the motion extent of external markers or the pressure in a belt or the variation in air flow. The latest techniques involve optical surface (OS) scanning systems such as the Sentinel™ and Catalyst™ (C‐rad Positioning AB, Uppsala, Sweden) or AlignRT (VisionRT, London, UK). The systems project light onto the patients’ skin surface and reconstruct a three‐dimensional surface of the patient. The OS system detects the patients’ position and movements and is used to trigger the beam for treatment delivery in DIBH.^19^ Several studies have evaluated patient setup accuracy during DIBH for left‐sided breast cancer for 3D surface matching,^20^, ^21^, ^22^ but few have investigated any dosimetric effects due to potential positioning deviations. For instance, Tang et al.^23^ evaluated the dosimetric impact of motion during DIBH for an iterative closest point (ICP)‐based algorithm for surface matching with the AlignRT system, using a ± 3 mm and ±3° tolerance for translational and rotational differences. They reported very small (<1 mm) breath‐hold motion and the dosimetric consequences were found to be small. However, only the rigid motion of the surface was investigated.

By using a novel nonrigid registration algorithm as well as finite element simulation of underlying tissues, the real‐time isocenter position can be determined from the surface motion. Thus, the optical surface scanning system (Catalyst™) can not only track the surface but also the real‐time isocenter position.^24^, ^25^ Several studies have shown dosimetric benefits of the DIBH treatment technique for breast cancer patients^13^, ^14^, ^15^ and several studies have shown increased accuracy in patient positioning using optical surface scanning.^20^, ^21^, ^22^ However, to the best of our knowledge, no investigation of potential dosimetric effects due to intrafractional isocenter motion during DIBH has been carried out and are thus highly desirable.

In this study, audio and visual guidance were used for the patients to achieve reproducible DIBHs. The surface over the xiphoid process worked as the surrogate for the target position for beam triggering during both CT imaging and treatment. During DIBH at the treatment machine, the Catalyst™ system was used for triggering the beam when the xiphoid process entered the gating window and simultaneously tracked the isocenter position. The intrafractional DIBH isocenter reproducibility in between breath holds was investigated and the subsequent dosimetric effects evaluated for both tangential and locoregional treatment of left‐sided breast cancer.

The aim of this study was to investigate potential dose reductions to organs at risk (OARs) using DIBH and optical surface scanning, and further evaluate how any dosimetric benefits are affected by possible intrafractional isocenter motion in between breath holds.

## 2 MATERIAL AND METHOD

### 2.A Ethical consideration and consent

The use of the radiotherapy database for retrospective research has been approved by the Regional Ethical Review Board in Lund (No. 2013/742).

### 2.B Patient selection

A total of 40 patients receiving radiotherapy for left‐sided breast cancer in DIBH were enrolled in this study, 20 patients received tangential treatment after breast‐conserving surgery and 20 patients received locoregional treatment after either breast‐conserving surgery or mastectomy. The patients started treatment between September 2015 and August 2016. The median age was 59 yr (range: 45–77 yr) for the group receiving tangential treatment and 46 yr (35–85 yr) for the group receiving locoregional treatment.

### 2.C Computed tomography simulations and treatment planning

All patients underwent supine computed tomography (CT) in separate scans for free breathing (FB) and DIBH. Images with a slice thickness of 3 mm were acquired using a Siemens Somatom definition AS plus (Siemens Medical Solutions, Erlangen, Germany). The prospective DIBH study was performed with the Sentinel™ system (C‐rad Positioning AB, Uppsala, Sweden) using laser (*λ* = 635–690 nm) to create a reference surface of the patient in FB and record the breathing motion.^26^ The patients were scanned with the Sentinel™ system in FB and a region of interest (ROI) was defined on the surface of the skin above xiphoid process in a shape of a circle with a diameter of 2 cm. Motion in the vertical direction in the ROI was registered as the breathing signal. Since the registered motion was in the vertical direction only, the rather flat and stable surface on sternum was chosen. The position of the end‐expiration for FB level, that is, the baseline, was automatically tracked and the amplitude was individually set for each patient at the maximum comfortable and reproducible breath hold level. The gating window was 3 mm for all patients included. Visual guidance with video goggles was used to help the patients keep the inspiration level in the gating window during the CT acquisition. To avoid abdominal breathing or so‐called fake breathing, that is, arching the back, the patients were asked to breathe with their chest during a short training session of a few breath holds using visual feedback prior to CT imaging. For patients receiving locoregional treatment after mastectomy, a bolus (Superflab, Mick Radio‐Nuclear Instruments, Inc. An Eckert & Ziegler BEBIG Company) was placed on the thoracic wall over the operation scar with a 3 cm margin at CT.

#### 2.C.1 Structure delineation

All structures were delineated in both the DIBH and FB CT sets by the same radiation oncologist, to reduce the interobserver variability. For tangential breast irradiation after breast‐conserving surgery, the PTV was defined as the clinical limits of the remaining breast including all glandular tissue. For cases receiving locoregional treatment after breast‐conserving surgery, ipsilateral axillary lymph nodes level II‐III and lymph nodes in the supra‐ and infraclavicular fossa were included in the PTV. Thus, the internal mammary nodes were not included. The CTV‐T was delineated as the tumor's position in the breast with at least 10 mm margin, approximately equivalent to a quadrant of the breast. After mastectomy, the PTV was defined as the part of the thoracic wall where the breast had been located (visualized on CT scans by markers), including ipsilateral lymph node stations as above. No CTV‐T was delineated for these patients. For all patients, the PTV was cropped 5 mm from the skin surface.

The OARs delineated were the heart, LAD, and the ipsilateral lung. The lung was automatically delineated using the segmentation wizard in the treatment planning system (TPS, Eclipse, version 13.6.30, Varian Medical Systems, Palo Alto, CA, USA) and then manually verified. The heart was defined as the entire myocardium including the large vessels up to the departure of the coronary arteries from aorta ascendens. LAD was delineated with a 6 mm diameter from the vessels departure from aorta as far as it could be visualized, often to the middle of the heart. The heart and LAD were delineated manually and all OARs were delineated without margins.

#### 2.C.2 Treatment planning

The treatment plans were created in the Eclipse TPS for an Elekta Synergy (Elekta AB, Stockholm, Sweden) and the dose was calculated using the anisotropic analytic algorithm (AAA, version 10.0.28 and 13.6.23). The prescribed dose was 50 Gy in 25 fractions, normalized to the PTV mean dose. All treatment plans were made by one dosimetrist, for the plans to be comparable between DIBH and FB. The main goal when creating the treatment plans was to fulfill the constraints presented in Table 1, based on national guidelines for 2014–2016 from the Swedish Breast Cancer Group (www.swebcg.se). At the same time, the OAR doses were kept as low as possible.

**Table 1:** Constraints aimed to be fulfilled when creating the treatment plans

Structure	Constraint
CTV‐T	V_95%_ = 100%
CTV‐T	D_mean_ ≥100%
PTV	D_98%_ ≥93%
PTV	V_105%_ minimized

Essentially identical three‐dimensional conformal radiotherapy (3D‐CRT) isocentric treatment plans were created for both DIBH and FB, where only minor differences in the beam arrangement were allowed to achieve comparable target coverage. For tangential treatment, two tangential opposing fields were used to irradiate the breast. If needed for dose homogenization, wedges and/or supplementary fields were used. The energy 6 MV was used for all fields. For locoregional treatment, 6 MV tangential opposing fields were used to irradiate the breast and, if necessary, supplementary fields were added (6 MV). The lymph nodes were irradiated using a 6 MV anterior–posterior (AP) field and a 10 MV posterior–anterior (PA) field. Additionally, a 10 MV PA field shielding the lung was added. For tangential treatment, the isocenter was placed in the center of the breast, and for locoregional treatment, the isocenter was placed in the junction between the tangential and AP/PA fields.

### 2.D Dosimetric comparison between DIBH and FB treatment plans

For the comparison between the DIBH and FB treatment plans, the mean dose to the heart (D_mean,heart_), LAD (D_mean,LAD_), and ipsilateral lung (D_mean,lung_), the dose received by 2% of the volume for the heart (D_2%,heart_) and LAD (D_2%,LAD_), the volume receiving 20 Gy for the ipsilateral lung (V_20Gy,lung_) and the dose received by 98% of the PTV volume (D_98%,PTV_) were retrieved from the TPS. Two‐sided paired Wilcoxon tests were carried out to investigate if the differences between DIBH and FB were statistically significant, using a significance level of 0.05.

### 2.E The DIBH treatment workflow using Catalyst™

When the treatment plan was finished, the plan isocenter, treatment fields, and plan UID were exported in DICOM format from the TPS to the Catalyst™ system. To assess the treatment isocenter for patient positioning in FB, the reference surface from the Sentinel™ system was connected to the isocenter of the treatment plan. The commissioning of the Catalyst™ system demonstrated an intrinsic accuracy of 0.2 mm isocenter localization at various isocenter depths confirmed by CBCT. The Catalyst™ system was used for the treatment sessions at the linear accelerator. The system uses a near invisible violet light (*λ* = 405 nm) projected onto the patient during surface imaging and measurement.^27^ The other wavelengths available, that is, green (*λ* = 528) and red (*λ* =624) lights, were used to project a color map onto the patient's surface indicating any setup deviations. The treatment workflow was divided into two modules, one positioning module and one treatment module. All patients were initially positioned in FB using a nonrigid algorithm for patient positioning.^24^, ^25^ The live surface of the patient being scanned on the treatment couch in FB was matched with the reference surface obtained with the Sentinel™ system at the CT session. Posture correction was manually performed with the help of the color map projected onto the patient's skin indicating deviations between the planned and real‐time position larger than 5 mm. The couch was shifted to the treatment position according to the calculations made by the OS system. A floating mean value filter over 4 s was used for the calculation to minimize the effect of the FB motion.

**Figure 1:**
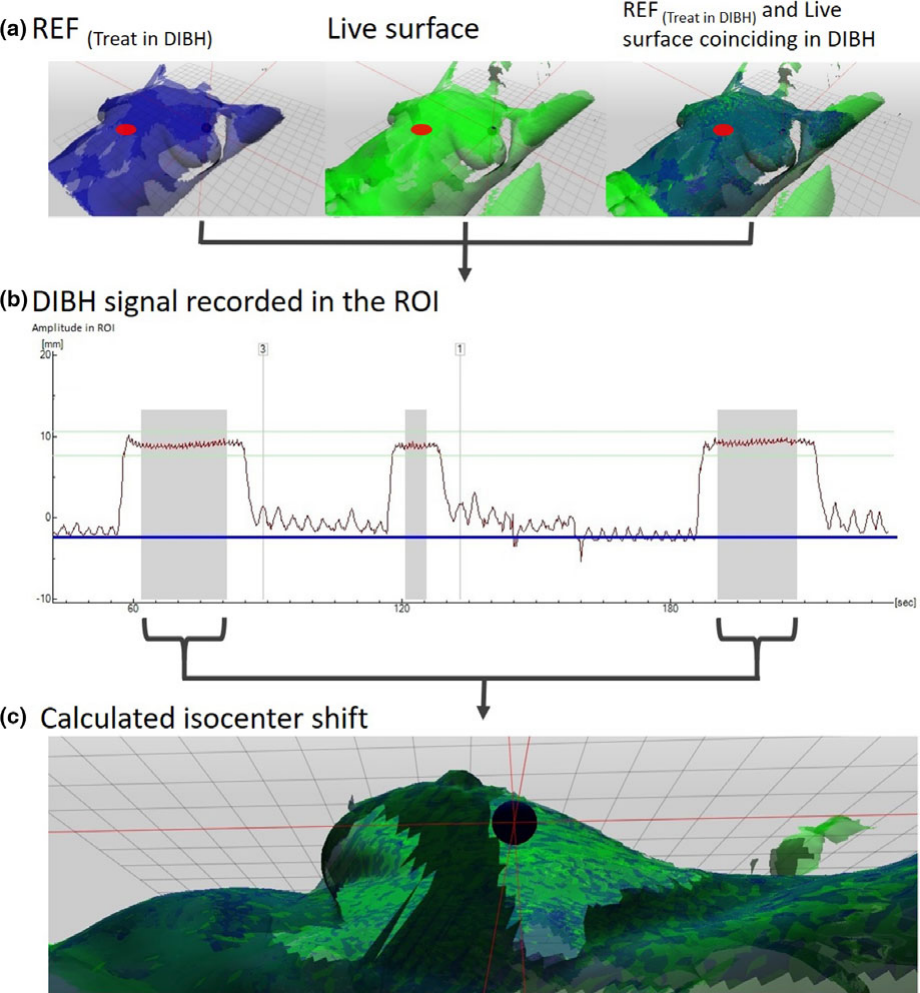
Workflow in the treatment module. The ROI shown as a red circle at xiphoid process was triggering beam‐on when the reference surface (REF_(treat in_ _DIBH__)_) was coinciding with the live surface (a). The DIBH signal in the ROI was tracked (b) and the isocenter shifts during beam‐on (gray fields in b) simultaneously recorded (c).

Once the patient was positioned correctly, the Catalyst™ treatment module was entered. The breathing baseline was established at the beginning of each treatment fraction, ensuring that the breathing amplitude was identical for every DIBH session, regardless of any daily residual setup deviation. Thus, the distance between the tumor volume and the heart could be maintained during irradiation, as well as the level of decreased lung density within the beam. If the setup needed to be corrected for prior to treatment, the baseline was recalculated. A daily surface reference image, REF_(Treat in DIBH)_, was captured the first time when the patient was breathing into the gating window. The live surface obtained during the rest of the treatment fraction was matched with REF_(Treat in DIBH)_, and hence these surfaces should coincide during DIBH [Fig. 1(a)]. This means that differences between the live surface and REF_(Treat in DIBH)_ do not include the residual setup errors, which are thus not transferred from the positioning module to the treatment module.

Two of the three algorithms provided by the Catalyst™ system with calculations for beam triggering for DIBH treatments were used in this study.^24^ The first algorithm was calculating the separation in z‐direction between the REF_(Treat in DIBH)_ and the live surfaces in an area predefined by the ROI. When the respiratory signal recorded in the ROI was within the gating window, the treatment beam was triggered [Fig. 1(b)]. Simultaneously, the Catalyst™ system's nonrigid algorithm was used to calculate any isocenter shifts by matching the REF_(Treat in DIBH)_ and the live surfaces [Fig. 1(c)]. However, no tolerances for beam triggering based on the isocenter shifts were set in this study and this was used for retrospective evaluation purposes only. Thus, the calculations within the ROI above the xiphoid process resulted in one‐dimensional breathing motion, presented to the patient by visual guidance, and triggered the beam within the narrow gating window. At the same time, the three‐dimensional motion data of the isocenter were passively collected.

### 2.F Assessment of the intrafractional DIBH isocenter reproducibility

In this study, the intrafractional DIBH isocenter reproducibility during beam‐on was investigated. The real time calculated isocenter position during beam‐on was obtained from the log files of the OS system. The intrafractional DIBH isocenter reproducibility was then calculated as the difference between the average isocenter positions during beam‐on for two DIBHs during each session. The two DIBHs were selected from the delivery of the two main fields, which represents the position for which the main part of the treatment was given. The intrafractional motion, that is, the relative shift between two DIBHs, was analyzed for five treatment sessions per patient. In total, 195 DIBHs per group were included in the analysis. One patient in each patient group was excluded due to treatment interruptions on the linear accelerator, causing the log files to be incomplete. For the tangential treatments, the isocenter shift between the two main fields were used and for the locoregional treatments, isocenter shifts from one of the tangential fields and one of the AP/PA fields were used. The intrafractional DIBH isocenter reproducibility in the lateral (lat), longitudinal (long), and vertical (vert) directions were analyzed separately for the two patient groups.

### 2.G Analysis of dosimetric effects induced by intrafractional DIBH isocenter reproducibility

The intrafractional DIBH isocenter reproducibility was applied to the treatment plans in the TPS as a set of isocenter shifts to generate dose distributions, resulting in an approximation of the dosimetric effects of the intrafractional isocenter DIBH motion. The intrafractional DIBH isocenter reproducibility in lat, long, and vert directions corresponding to a cumulative probability of 50% of the DIBHs, 90% of the DIBHs and the maximum (max) value, was used for the two patient groups. The cumulative probability means that for X% of the DIBHs, the intrafractional DIBH isocenter reproducibility is less or equal to Y mm. For example, the 50% cumulative probability represents the median isocenter reproducibility for the whole patient group. For each shift, the dose was recalculated in the original DIBH plan which, since all combination of directions were simulated, resulted in eight plans per shift level (50%, 90%, and max). In total, 960 isocenter‐shifted plans were obtained. For each isocenter‐shifted plan, the dose was recalculated keeping the same number of monitor units. From the resulting DVHs, the D_mean,heart_, D_mean,LAD,_ D_mean,lung_, D_2%,heart_, D_2%,LAD_, V_20Gy,lung_, and D_98%,PTV_ were obtained and the minimum and maximum values for each patient and probability level were considered for the motion‐induced dose effect evaluation.

## 3 RESULTS

### 3.A Dosimetric comparison between DIBH and FB treatment plans

**Figure 2:**
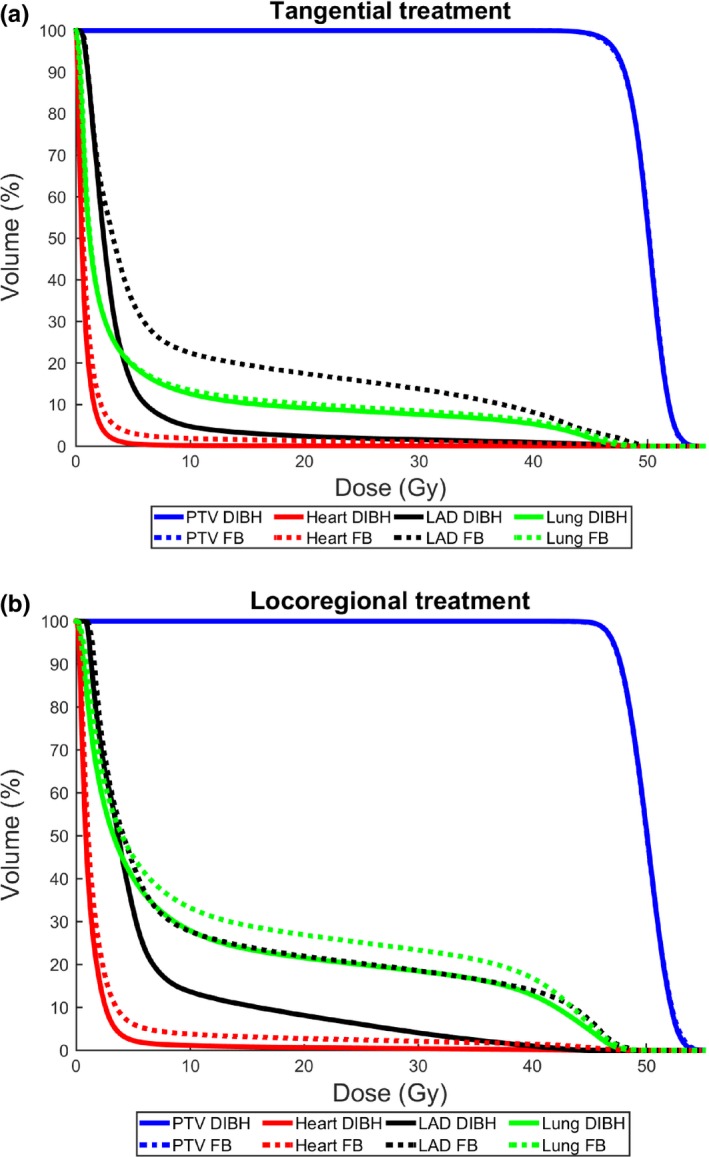
Average relative dose volume histograms for tangential (a) and locoregional (b) treatment for the heart (red), LAD (black), ipsilateral lung (green), and PTV (blue) comparing DIBH (solid lines) and FB (dashed lines).

**Figure 3:**
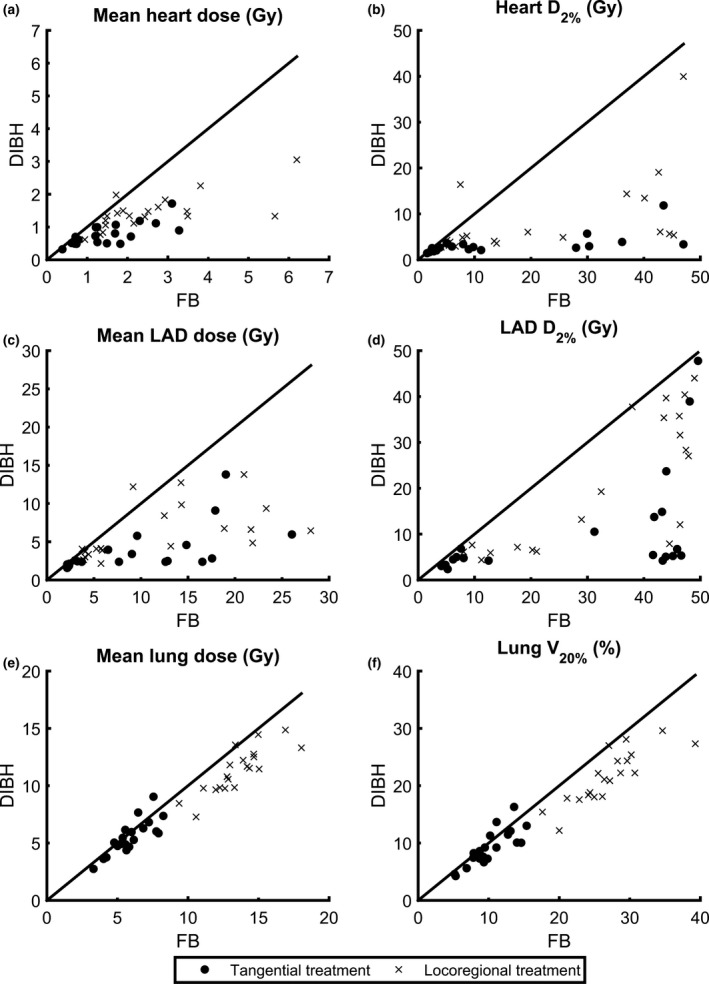
Individual values for each patient of D_mean,heart_ (a), D_2%,heart_ (b), D_mean,_ _LAD_ (c), D_2%,_ _LAD_ (d), D_mean,lung_ (e), and V_20Gy,lung_ (f) for deep inspiration breath‐hold (DIBH) versus free breathing (FB), for both tangential (circles) and locoregional (crosses) treatment. The lines are for illustration purpose only, and represent where the dosimetric parameters are equal for DIBH and FB. Hence, for points below the line, DIBH is superior to FB and for points above the line, FB is superior to DIBH.

Overall, the dose to the heart, LAD, and ipsilateral lung was reduced for all dose levels with comparable target coverage for DIBH compared to FB for both tangential and locoregional treatment [Fig. 2]. The heart and LAD mean doses and D_2%_ were reduced for essentially all patients using DIBH and the mean lung dose and V_20Gy_ were reduced for the majority of the patients [Fig. 3]. For patients with high OAR doses in FB, larger reductions of the dosimetric parameters were generally observed with DIBH [Fig. 3]. The median deep inspiration amplitude during CT, measured with the Sentinel™ system at the xiphoid process, was 10.5 mm (range: 5.4–19.6 mm) for tangential treatment and 10.3 mm (8.2–13.0 mm) for locoregional treatment.

For tangential treatment, the median mean heart and LAD doses were reduced by 44% (1.25 to 0.71 Gy, *P* < 0.001) and 70% (8.35 to 2.47 Gy, *P* < 0.001) for DIBH compared to FB (Table 2). Regarding near‐maximum doses, the D_2%_ to the heart and LAD were reduced by 61% (6.98 to 2.70 Gy, *P* < 0.001) and 87% (41.72 to 5.27 Gy, *P* < 0.001). For the ipsilateral lung, the median mean dose and V_20Gy_ were reduced from 5.74 to 5.36 Gy (*P* = 0.044) and from 9.66% to 8.92% (*P* = 0.025), respectively using DIBH, corresponding to relative reductions of 6% and 8%.

**Table 2:** Comparison of dosimetric parameters between DIBH and FB for tangential and locoregional treatment, presented as median values [range] and *P*‐values for paired Wilcoxon tests

	Tangential treatment			Locoregional treatment		
	FB	DIBH	*P*	FB	DIBH	*P*
D_mean,heart_	1.25 [0.39–3.28]	0.71 [0.32–1.72]	<0.001^a^	2.10 [0.94–6.20]	1.34 [0.62–3.05]	<0.001^a^
D_2%,heart_	6.98 [1.61–47.00]	2.70 [1.45–11.85]	<0.001^a^	13.68 [4.08–47.01]	5.05 [2.72–39.95]	<0.001^a^
D_mean,LAD_	8.35 [2.15–26.05]	2.47 [1.58–13.81]	<0.001^a^	10.82 [3.75–28.06]	4.63 [2.15–13.81]	<0.001^a^
D_2%,LAD_	41.72 [4.14–49.59]	5.27 [2.38–47.77]	<0.001^a^	40.74 [8.06–48.96]	16.22 [4.38–44.00]	<0.001^a^
D_mean,lung_	5.74 [3.30–8.26]	5.36 [2.75–9.05]	0.044^a^	13.32 [9.37–18.03]	11.49 [7.29–14.86]	<0.001^a^
V_20Gy,lung_	9.66 [5.17–15.39]	8.92 [4.25–16.30]	0.025^a^	26.72 [17.63–39.28]	21.63 [12.18–29.60]	<0.001^a^
D_98%,PTV_	92.06 [90.47–92.94]	92.47 [90.58–94.64]	0.117	93.07 [91.54–93.82]	92.97 [91.48–93.86]	0.433

a *Statistical significant difference (*P* < 0.05).

For locoregional treatment, the median mean heart and LAD doses were reduced by 36% (2.10 to 1.34 Gy, *P* < 0.001) and 57% (10.82 to 4.63 Gy, *P* < 0.001) for DIBH compared to FB (Table 2). Regarding near‐maximum doses, the D_2%_ to the heart and LAD were reduced by 63% (13.68 to 5.05 Gy, *P* < 0.001) and 60% (40.74 to 16.22 Gy, *P* < 0.001). For the ipsilateral lung, the median mean dose and V_20Gy_ were reduced from 13.32 to 11.49 Gy (*P* < 0.001) and from 26.72% to 21.63% (*P* < 0.001), respectively using DIBH, corresponding to relative reductions of 14% and 19%.

### 3.B Assessment of the intrafractional DIBH isocenter reproducibility

**Figure 4:**
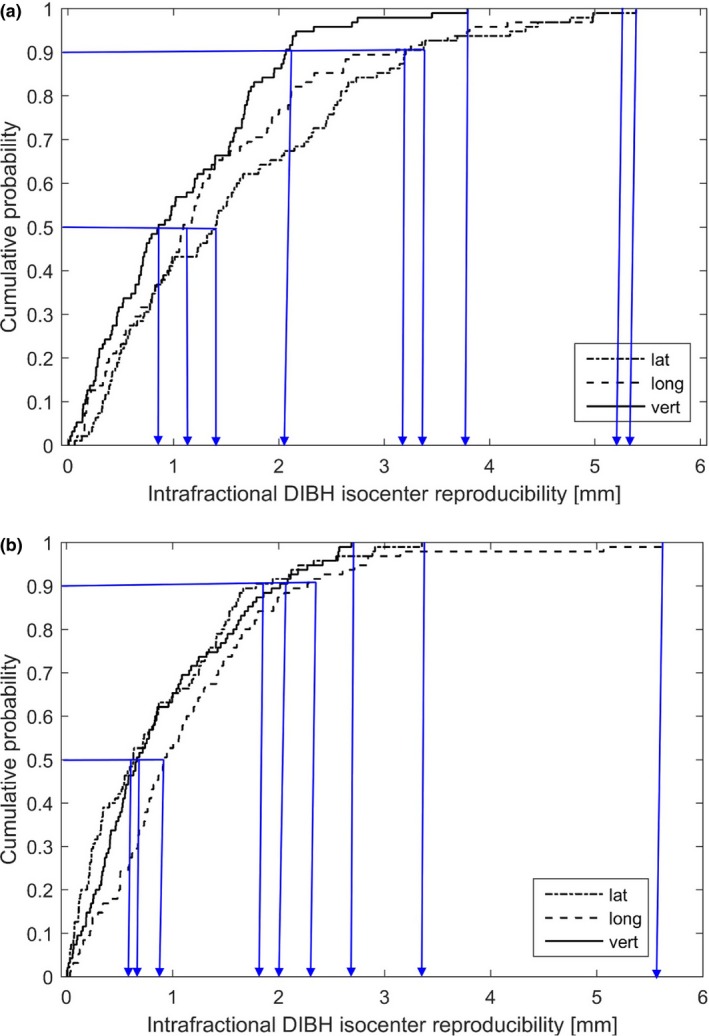
Cumulative probability of the intrafractional DIBH isocenter reproducibility for the tangential treatment (a) and locoregional treatment (b). The intrafractional DIBH isocenter reproducibility in lat, long, and vert directions corresponding to the cumulative probability of 50%, 90%, and maximum value are marked with blue arrows.

The cumulative probability of having an intrafractional DIBH isocenter reproducibility less or equal to a certain value was calculated in the lat, long, and vert directions and are presented for the two patient groups in Fig. 4. For tangential treatment, 50%/90% cumulative probabilities of having intrafractional DIBH isocenter reproducibility less or equal to 1.4/3.2, 1.1/3.1, and 0.9/2.1 mm in the lat, long, and vert directions, respectively, were observed (Fig. 4). The corresponding values for locoregional treatment were 0.6/1.8, 0.9/2.3, and 0.7/2.0 mm. The maximum values for the intrafractional DIBH isocenter reproducibility were 5.4, 5.3, and 3.8 mm (lat, long, and vert) for tangential treatment and 3.4, 5.6, and 2.7 mm for locoregional treatment (Fig. 4).

### 3.C Analysis of dosimetric effects induced by intrafractional DIBH isocenter reproducibility

**Figure 5:**
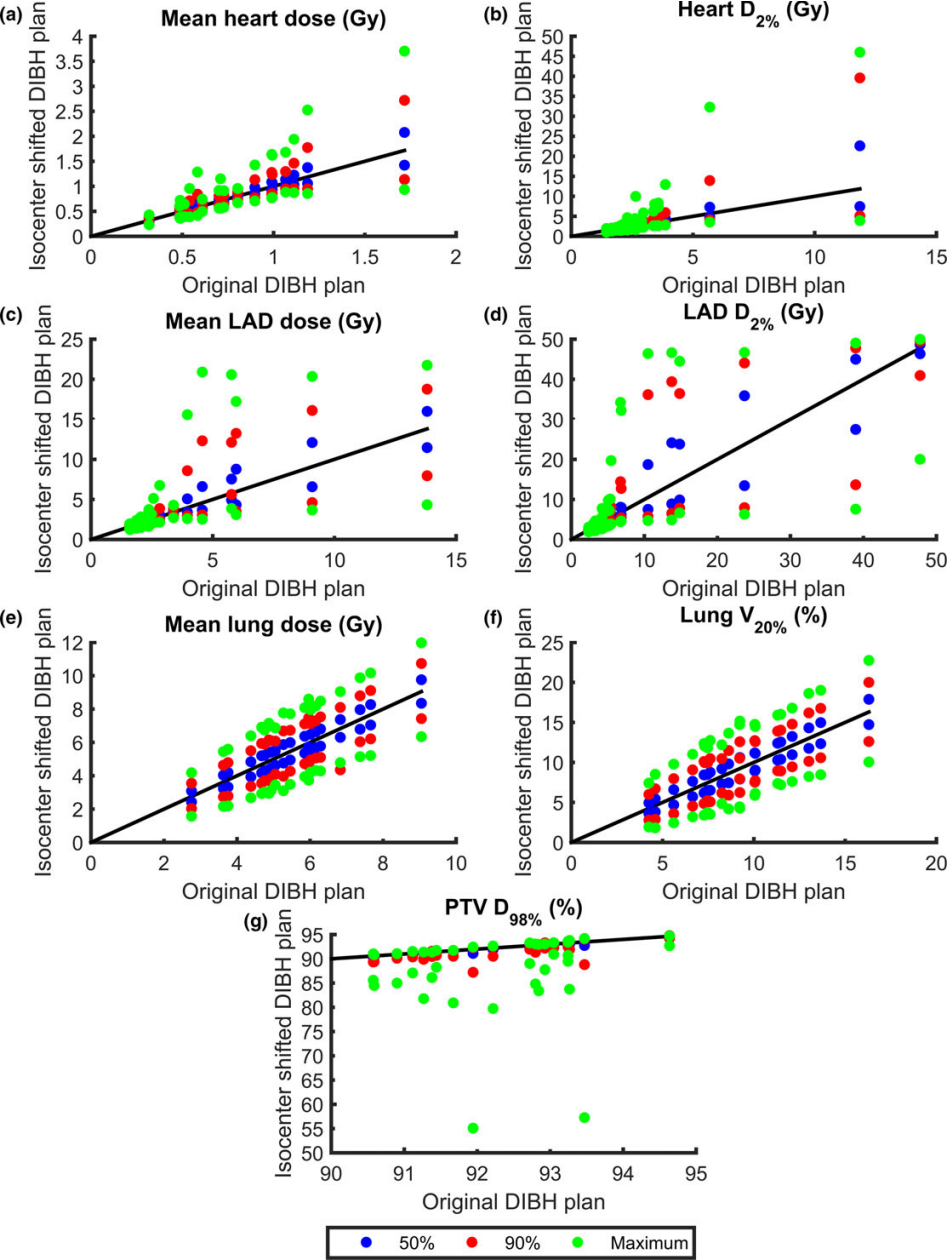
The minimum and maximum values of D_mean,heart_ (a), D_2%,heart_ (b), D_mean,_ _LAD_ (c), D_2%,_ _LAD_ (d), D_mean,lung_ (e), V_20Gy,lung_ (f), and D_98%,_ _PTV_ (g) for the isocenter‐shifted DIBH plans versus the original DIBH plans. The results are presented for each individual patient receiving tangential treatment and for all three cumulative probability levels (50%, 90%, and maximum). The lines are for illustration purpose only, and represent where the dosimetric parameters are equal for the isocenter‐shifted and original DIBH plans.

**Figure 6:**
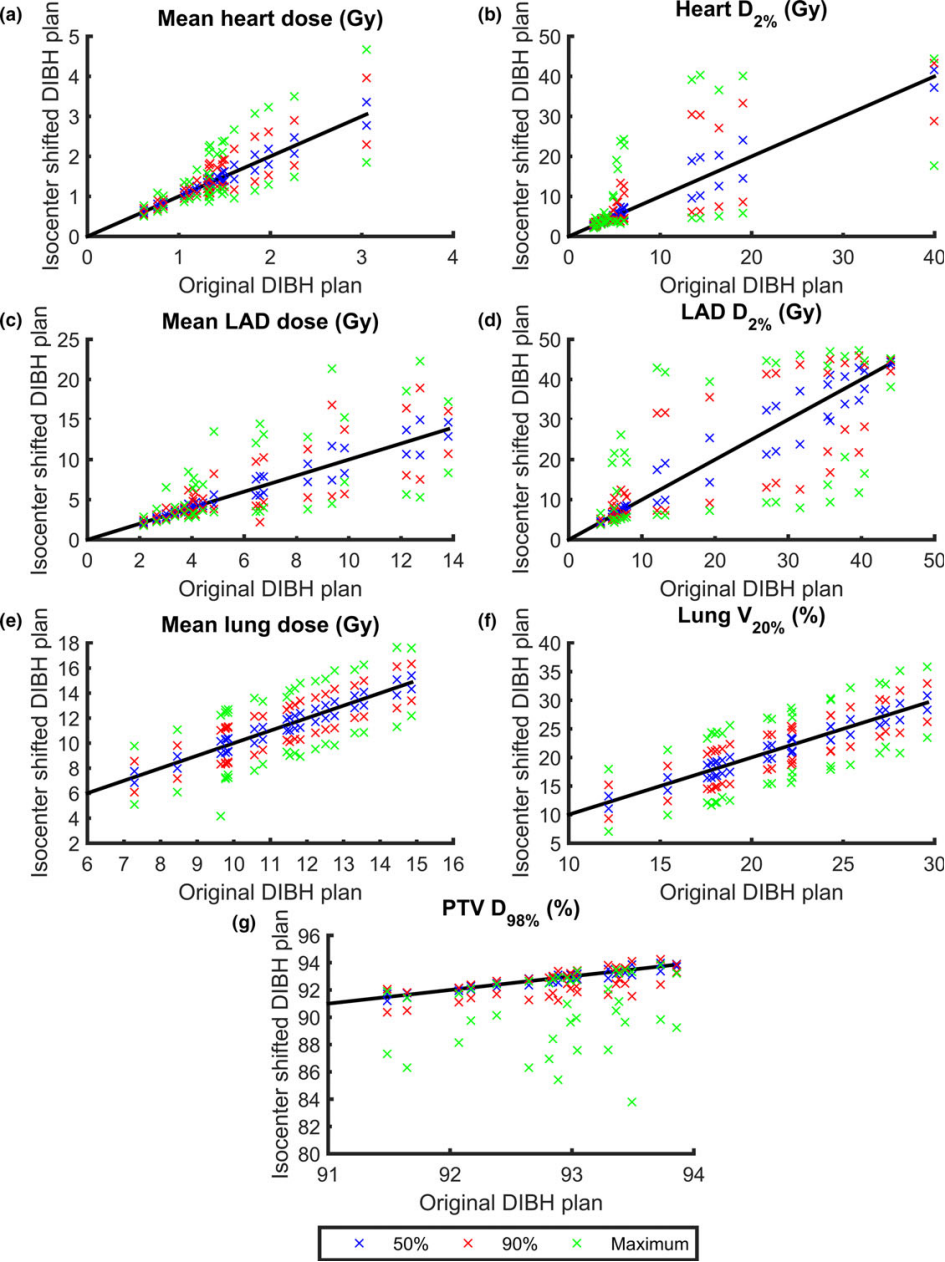
The minimum and maximum values of D_mean,heart_ (a), D_2%,heart_ (b), D_mean,_ _LAD_ (c), D_2%,_ _LAD_ (d), D_mean,lung_ (e), V_20Gy,lung_ (f), and D_98%,_ _PTV_ (g) for the isocenter‐shifted DIBH plans versus the original DIBH plans. The results are presented for each individual patient receiving locoregional treatment and for all three cumulative probability levels (50%, 90%, and maximum). The lines are for illustration purpose only, and represent where the dosimetric parameters are equal for the isocenter‐shifted and original DIBH plans.

The dosimetric effect of the intrafractional DIBH isocenter reproducibility is presented in Table 3 and Fig. 5 for tangential treatment and in Table 4 and Fig. 6 for locoregional treatment. Large interpatient variability in the dosimetric effect of the intrafractional DIBH isocenter reproducibility was observed, for both the PTV and OARs.

**Table 3:** The dosimetric effect of the intrafractional DIBH isocenter reproducibility for tangential treatment. The median values and ranges for the minimum and maximum values of the various dosimetric parameters for the three cumulative probability levels: 50%, 90%, and maximum (M) are presented for the isocenter‐shifted plans. The corresponding values for the original DIBH plans are included for comparison

	Original plan	Isocenter shifted plan					
		50%_min_	50%_max_	90%_min_	90%_max_	M_min_	M_max_
D_mean,heart_	0.71 [0.32–1.72]	0.67 [0.30–1.42]	0.75 [0.34–2.08]	0.62 [0.27–1.14]	0.84 [0.38–2.72]	0.56 [0.23–0.94]	0.96 [0.43–3.71]
D_2%,heart_	2.70 [1.45–11.85]	2.50 [1.35–7.46]	3.00 [1.56–22.60]	2.31 [1.21–5.10]	3.37 [1.73–39.61]	2.09 [1.05–3.92]	4.54 [1.99–46.03]
D_mean,LAD_	2.47 [1.58–13.81]	2.34 [1.51–11.48]	2.61 [1.65–16.01]	2.18 [1.40–7.94]	2.92 [1.76–18.76]	1.99 [1.25–4.34]	3.67 [1.92–21.75]
D_2%,LAD_	5.27 [2.38–47.77]	4.84 [2.27–46.34]	5.79 [2.51–48.68]	4.31 [2.10–40.93]	6.83 [2.71–49.47]	3.71 [1.93–20.00]	9.93 [3.06–49.97]
D_mean,lung_	5.36 [2.75–9.05]	4.85 [2.43–8.34]	5.79 [3.09–9.76]	4.18 [2.03–7.43]	6.71 [3.55–10.73]	3.46 [1.57–6.34]	7.74 [4.18–11.97]
V_20Gy,lung_	8.92 [4.25–16.30]	7.67 [3.60–14.73]	10.17 [4.96–17.89]	6.21 [2.81–12.62]	11.01 [5.99–20.02]	4.37 [1.82–10.06]	14.08 [7.41–22.75]
D_98%,PTV_	92.47 [90.58–94.64]	92.19 [30.35–94.46]	92.64 [90.66–94.73]	90.61 [87.23–94.24]	92.72 [90.73–94.79]	85.29 [55.08–92.69]	92.81 [90.83–94.76]

**Table 4:** The dosimetric effect of the intrafractional DIBH reproducibility for locoregional treatment. The median values and ranges for the minimum and maximum values of the various dosimetric parameters for the three cumulative probability levels: 50%, 90%, and maximum (M) are presented for the isocenter‐shifted plans. The corresponding values for the original DIBH plans are included for comparison

	Original plan	Isocenter‐shifted plan					
		50%_min_	50%_max_	90%_min_	90%_max_	M_min_	M_max_
D_mean,heart_	1.34 [0.62–3.05]	1.29 [0.59–2.77]	1.48 [0.65–3.35]	1.18 [0.55–2.30]	1.75 [0.70–3.96]	1.03 [0.50–1.85]	2.10 [0.77–4.67]
D_2%,heart_	5.05 [2.72–39.95]	4.65 [2.59–37.16]	5.58 [2.86–41.63]	4.11 [2.36–28.81]	7.59 [3.13–43.28]	3.75 [2.12–17.66]	13.55 [3.48–44.37]
D_mean,LAD_	4.63 [2.15–3.81]	4.29 [2.07–12.90]	5.15 [2.24–14.95]	3.89 [1.90–10.74]	6.03 [2.19–18.93]	3.48 [1.75–8.35]	10.27 [2.86–22.28]
D_2%,LAD_	16.22 [4.38–44.00]	12.13 [4.24–43.55]	22.24 [4.60–44.38]	8.23 [4.01–42.09]	33.64 [5.22–45.90]	6.74 [3.79–38.11]	42.37 [6.62–47.25]
D_mean,lung_	11.49 [7.29–14.86]	11.01 [6.84–14.33]	11.97 [7.74–15.40]	10.18 [6.08–13.39]	12.82 [8.57–16.33]	8.97 [4.16–12.18]	13.94 [9.77–17.64]
V_20Gy,lung_	21.63 [12.18–29.60]	20.32 [11.03–28.33]	22.66 [13.25–30.80]	18.39 [9.33–26.24]	24.40 [15.18–32.91]	15.53 [7.04–23.45]	26.97 [17.99–35.84]
D_98%,PTV_	92.97 [91.48–93.86]	92.79 [91.20–93.72]	93.10 [91.72–93.98]	91.74 [90.36–93.30]	93.15 [91.82–94.25]	88.83 [83.80–91.15]	92.78 [91.42–93.85]

The minimum values of the PTV D_98%_ were always observed when the isocenter shifts were applied in the left, cranial and anterior directions, since the combination of these directions correspond to the maximum movement of the PTV out of the treatment fields. For tangential treatment, the median values of the minimum D_98%,PTV_ decreased from 92.47% in the original DIBH plan to 92.19, 90.61, and 85.29% when isocenter shifts corresponding to the 50%, 90%, and maximum cumulative probabilities were applied (Table 3). For locoregional treatment, the corresponding values decreased from 92.97% to 92.79, 91.87, and 88.83% (Table 4).

For all OARs, the maximum values of the dosimetric parameters were always observed when the isocenter shifts were applied in the right, caudal and posterior directions, since the combination of these directions correspond to the maximum movement of the OARs into the treatment fields. Correspondingly, the minimum values of the dosimetric parameters for the OARs were always observed when the isocenter shifts were applied in the left, cranial and anterior directions, since this combination of directions correspond to a maximum separation of the OARs and the treatment fields. For example, for tangential treatment, the median values of the maximum D_2%,heart_ were increased from 2.70 Gy in the original DIBH plan to 3.00, 3.37, and 4.54 Gy when isocenter shifts corresponding to the 50%, 90%, and maximum cumulative probabilities were applied (Table 3). The corresponding values for D_2%,LAD_ increased from 5.27 Gy to 5.79, 6.83, and 9.93 Gy and the median value of the maximum V_20Gy,lung_ increased from 8.92 Gy to 10.17, 11.01, and 14.08 Gy. For locoregional treatment, the median values of the maximum D_2%,heart_ were increased from 5.05 Gy in the original DIBH plan to 5.58, 7.59, and 13.55 Gy when the isocenter shifts corresponding to the 50%, 90%, and maximum cumulative probabilities were applied (Table 4). The corresponding values for D_2%,LAD_ increased from 16.22 Gy to 22.24, 33.64, and 42.37 Gy and the median value of the maximum V_20Gy,lung_ increased from 21.63 Gy to 22.66, 24.40, and 26.97 Gy.

## 4 DISCUSSION

This study showed that using the Catalyst™ system for DIBH treatments with visual guidance significantly reduces both the mean dose to the heart and LAD and high dose volumes, for both tangential and locoregional treatment (Fig. 2). Also, the dose to the ipsilateral lung could be reduced. This may reduce the risk of long‐term cardiovascular and pulmonary mortality and morbidity. The heart and LAD dose reductions observed in this study using DIBH was comparable to the dose reductions previously observed.^13^, ^14^, ^15^ Smyth et al.^14^ reviewed ten treatment planning studies comparing DIBH and FB, all showing a significant reduction of the mean heart and LAD dose using DIBH. The relative reduction in the mean dose was between 38% and 65% for the heart and between 31% and 71% for LAD. The relative reductions in the mean heart dose observed in our study (44% and 36%) were in the lower part or slightly below the range presented by Smyth et al. This could, however, be explained by the fact that the absolute mean heart dose in both FB and DIBH were lower in our study compared to all studies included in the review. For the mean LAD doses, the relative reductions observed in our study (70% and 57%) were within or slightly larger than the range presented by Smyth et al.^14^ However, also the mean LAD doses presented in our study were lower than the smallest values presented by Smyth et al.^14^ In a large systematic review of cardiac doses by Taylor et al.,^13^ it was shown that when the internal mammary node was not included in the target, the average mean dose to the heart could be reduced from 3.8 Gy in FB to 1.3 Gy using DIBH. Also, compared to that review, our study generally demonstrated lower mean heart doses. In the review by Smyth et al.,^14^ there was a large variety in heart and LAD doses between the different studies, which may, for example, depend on variations in the delineation of the target and OARs and the treatment technique used, making it difficult to compare the doses from the different studies. To reduce the interobserver variability in this study, all structures were delineated by the same oncologist and all treatment plans were created by the same dosimetrist, minimizing the uncertainties in the dosimetric comparison between FB and DIBH as much as possible.

Comparing the results of this study and our previous study investing the benefits of enhanced inspiration gating (EIG),^15^ lower relative reductions of the doses to the heart and LAD could generally be observed (except for the LAD D_2%_), which is likely because overall higher absolute doses were observed in the previous study. The reason for this may be differences in the delineations of the structures and the creation of the treatment plans, since these tasks were carried out by different physicians and dosimetrists in the two studies. A significant reduction in the lung dose for tangential treatment was observed for DIBH in this study, which was not seen for EIG in our previous study. This was also observed by Damkjær et al.^28^ comparing DIBH and EIG, and is probably due to the higher breathing amplitude achieved using DIBH.

In a study by Chung et al.,^29^ 32 patients underwent cardiac SPECT‐CT before and after left‐sided breast cancer radiotherapy, where no part of the heart was allowed inside the treatment beams. No perfusion defects were observed, which has been seen in previous studies where parts of the heart were located inside the treatment fields.^30^ This may indicate that it is the inclusion of the heart in the primary beam that is of concern. It is therefore of importance to remove the entire heart from the primary beam, shown to be possible using DIBH. In this study, the number of patients with the heart completely outside the treatment fields was increased from 4 for FB to 16 for DIBH for tangential treatment and from 0 for FB to 9 for DIBH for locoregional treatment.

In this study, we have shown that it is possible to reduce OAR doses using the Catalyst™ system for DIBH treatments with visual guidance, but when using this technique, it is of utmost importance not to introduce motion‐induced uncertainties during the treatment delivery. We have, therefore, assessed and estimated the dosimetric effect of intrafractional DIBH isocenter reproducibility for the two patient groups, using real‐time tracking of the isocenter position during the treatment delivery. The intrafractional DIBH isocenter reproducibility was found to be very good for the majority of the treatment sessions observed in this study, with a typical median value around 1 mm (Fig. 4). These results are in the same order as reported previously from similar studies, showing discrepancies of approximately 2 mm.^20^, ^22^ In these studies, however, the surface was used as a surrogate during DIBH, and hence, the isocenter position was not investigated. However, for a few occasional treatment sessions in this study, the intrafractional DIBH isocenter reproducibility was found to be approximately 5 mm, which resulted in large effects on the target coverage and OARs doses (Tables 3 and 4, Figs. 5 and 6). However, reduced OAR doses were maintained compared to FB in most cases, with some exceptions observed for the maximum isocenter shifts. There is also motion during FB, but this has not been taken into account in this study. Despite only allowing beam‐on within a 3 mm gating window based on the movement of the xiphoid process, larger differences in the isocenter position between two different DIBHs were observed, in either of the three translational directions, for 16 patients and 26 treatment sessions in total. This implies that the motion of the target volume differs from the xiphoid process, used to trigger the beam. Hence, it is of importance not to only perform DIBH based on the bony anatomy of the xiphoid process but also set tolerance levels on the isocenter position. Using the nonrigid algorithm, the Catalyst™ system provides the possibility to set tolerances on the allowed isocenter shift and rotation, which would be more representative of the target position. Then large isocenter shifts with the associated dosimetric impact shown in our study could be avoided. For example, using the results from our study, it can be observed that using the same tolerance of 3 mm as for xiphoid process for the isocenter shift in lat, long, and vert direction, respectively, would result in isocenter tolerance failure in 1.0%/3.2%/0.0% of the treatment sessions for locoregional treatment, and in 14.7%/10.5%/2.1% of the treatment sessions for tangential treatments (Fig. 4).

Worse intrafractional DIBH isocenter reproducibility was observed for tangential treatment compared to locoregional treatment, probably due to the different positioning of the isocenter. For tangential treatment, the isocenter was positioned in the center of the breast, whereas for the locoregional treatment, the isocenter was positioned in the junction between the breast and the AP/PA fields. The breast is more deformable, while the junction between breast and the AP/PA fields is a more rigid structure, and therefore, the two isocenter positions move differently relative to the xiphoid process. However, the breast tissue is also a part of the PTV for the locoregional treatment. The time between the two DIBHs could potentially also be a reason for the difference in reproducibility. However, this was found to be similar for the two patient groups as the median time between the two analyzed DIBHs were 2.5 (range: 0.6–8.0) min and 2.3 (0.5–9.5) min for tangential and locoregional treatment, respectively.

Large interpatient variability in the dosimetric effect was observed (Figs. 5 and 6), especially for heart and LAD, due to differences in the patient anatomy and the placement of the treatment fields relative to the target volume and OARs in the original plan. For some patients, the heart and LAD were well out of the treatment fields in the original DIBH plan. For these cases, the applied isocenter shifts did not bring the heart and LAD into the treatment fields and hence only small dosimetric effects were observed. Similarly, for some patients, a large part of the heart and LAD was already inside the treatment fields in the original DIBH plan and the applied isocenter shifts did not bring the heart and LAD out of the treatment fields. The largest dosimetric effects were observed for the patients where the treatment field edges were contiguous with the edge of the heart and LAD, since for these patients, the applied isocenter shifts would either bring the heart and LAD into or out of the treatment fields. This effect was most pronounced for D_2%_, since this represents the near‐maximum dose. In Figs. 5 and 6, it can also be observed that the dosimetric effect of the applied isocenter shifts is not symmetrical for D_98%,PTV_. Underdosage is more common than overdosage, since target coverage will remain (but not increase) if the isocenter shifts result in the treatment fields being located too deep. However, if the treatment fields are too shallow, the D_98%,PTV_ will decrease rapidly, resulting in an underdosage of the target volume. To reduce the OAR and target volume dose deviations for individual treatment sessions, it would be of great importance to introduce tolerances for the isocenter deviation, which could, for example, correspond to the 90% cumulative probability level. If such tolerance is applied, the results from this study show that the dose deviations for the maximum and 90% cumulative probability level compared to the original plan could be reduced from 35% to 18% for the median D_mean,heart_, 68% to 25% for the median D_2%,heart_, 49% to 18% for the median D_mean,LAD_, and 88% to 30% for the median D_2%,LAD_ for tangential treatment (Table 3). The corresponding values for locoregional treatment were 57% to 31% for the median D_mean,heart_, 168% to 50% for the median D_2%,heart_, 122% to 30% for the median D_mean,LAD_, and 161% to 107% for the median D_2%,LAD_ (Table 4). Also, the minimum deviations in the median D_98%,PTV_ could be reduced from 8% to 2% and from 4% to 1% for tangential and locoregional treatment, respectively (Tables 3 and 4).

One limitation of this study is that the dosimetric effect of the intrafractional DIBH isocenter reproducibility was estimated using isocenter shifts in the TPS. When performing isocenter shifts a rigid motion is assumed, that is, the whole patient moves the corresponding isocenter shift and the distance between the heart and target volume is thus kept constant. This is actually not true since the distance between the heart and target volume changes with breathing. Using a deformable patient model would probably improve the accuracy of these calculations. But since the isocenter shifts were rather small (in the order of a few millimeter), we believe our calculations still gives a reasonable approximation of the dosimetric effect.

The analysis of this study was population based, using the cumulative probability of the intrafractional DIBH isocenter reproducibility to simulate the dosimetric effects for each patient plan. Hence, the dosimetric effect of each patient's individual isocenter reproducibility was not simulated. The maximum intrafractional DIBH isocenter reproducibility represents the worst‐case scenario for the entire population and deviations of this magnitude were only observed for a few percent of the DIBHs. The results in this study are based on the assumption that the isocenter reproducibility was the same for every DIBH, which of course is not the case. Slightly different isocenter positions will be obtained for each DIBH throughout the treatment, resulting in a small blurring of the dose distribution. The total delivered dose to the patient would, therefore, most likely differ less from the planned dose than the result presented in this study. The 50% cumulative probability level would probably be a more realistic representation of the dosimetric effects caused by the intrafractional DIBH isocenter reproducibility for an entire treatment.

Overall, using the xiphoid process as a surrogate for the breast tissue during DIBH was found to be reproducible (Fig. 4). Gierga et al.^22^ reported that 22% of the DIBHs were out of a 5 mm tolerance using the breast surface to trigger the beam when a rigid match algorithm and audio coaching were used. If using a 5 mm tolerance in either lat, long, or vert direction in this study, only 2% of the DIBH would be out of tolerance for locoregional treatments and 1% for tangential treatments. This implies that using the xiphoid process as a surrogate for the breast tissue improved the intrafractional DIBH isocenter reproducibility compared to using the breast surface. And according to this study, the intrafractional DIBH isocenter reproducibility could be improved even further by introducing tolerances on the isocenter position.

## 5 CONCLUSION

Deep inspiration breath‐hold (DIBH) treatments for breast cancer radiotherapy, using the optical surface scanning system Catalyst™ including visual guidance, reduces the absorbed doses to the heart, LAD, and ipsilateral lung in accordance to previous studies, which may reduce the risk of long‐term cardiovascular and pulmonary mortality and morbidity.

Excellent intrafractional DIBH isocenter reproducibility was observed for the majority of the treatment sessions for both tangential and locoregional treatment. However, values of the intrafractional DIBH isocenter reproducibility up to approximately 5 mm were seen for some treatment sessions, which resulted in large dosimetric effects, primarily for the OARs. Hence, it is of importance to set tolerance levels on the intrafractional isocenter motion and not only perform DIBH based on the motion of the bony anatomy of the xiphoid process.

## CONFLICT OF INTEREST

All authors approved the final manuscript, and declared that they have no potential conflicts of interest to this work.

## DECLARATION OF INTEREST

Our research is partly financially supported by C‐RAD AB, Uppsala, Sweden, however, the authors alone are responsible for the content, analyses, and writing.
